# Ferulic Acid Orchestrates Anti-Oxidative Properties of Danggui Buxue Tang, an Ancient Herbal Decoction: Elucidation by Chemical Knock-Out Approach

**DOI:** 10.1371/journal.pone.0165486

**Published:** 2016-11-08

**Authors:** Amy G. W. Gong, Vincent Y. Huang, Huai Y. Wang, Huang Q. Lin, Tina T. X. Dong, Karl W. K. Tsim

**Affiliations:** 1 Division of Life Science and Center for Chinese Medicine, The Hong Kong University of Science and Technology, Clear Water Bay, Hong Kong, China; 2 HKUST Shenzhen Research Institute, Hi-Tech Park, Nanshan, Shenzhen, 518000, Guangdong Province, China; Suzhou University, CHINA

## Abstract

Ferulic acid, a phenolic acid derived mainly from a Chinese herb Angelica Sinensis Radix (ASR), was reported to reduce the formation of free radicals. Danggui Buxue Tang (DBT), a herbal decoction composing of Astragali Radix (AR) and ASR, has been utilized for more than 800 years in China having known anti-oxidative property. Ferulic acid is a major active ingredient in DBT; however, the role of ferulic acid within the herbal mixture has not been resolved. In order to elucidate the function of ferulic acid within this herbal decoction, a ferulic acid-depleted herbal decoction was created and named as DBT_Δfa_. The anti-oxidative properties of chemically modified DBT decoction were systemically compared in cultured H9C2 rat cardiomyoblast cell line. The application of DBT and DBT_Δfa_ into the cultures showed functions in (i) decreasing the reactive oxygen species (ROS) formation, detected by laser confocal; (ii) increasing of the activation of Akt; (iii) increasing the transcriptional activity of anti-oxidant response element (ARE); and (iv) increasing the expressions of anti-oxidant enzymes, i.e. NQO1 and GCLM. In all scenario, the aforementioned anti-oxidative properties of DBT_Δfa_ in H9C2 cells were significantly reduced, as compared to authentic DBT. Thus, ferulic acid could be an indispensable chemical in DBT to orchestrate multi-components of DBT as to achieve maximal anti-oxidative functions.

## Introduction

Cardiovascular illnesses are the leading cause of death, worldwide. Over 1,750 million people are estimated to be died of cardiovascular illnesses, accounting for about 30% of global deaths per year [1]. Under aerobic stages, the production of reactive oxygen species (ROS) has a closely relationship with cellular damages [2]. ROS is able to cause the damage of macromolecules in cells, and therefore ROS is thought to be implicated in pathogenesis of various diseases, including cardiovascular problems. Moreover, ROS participates as a benevolent molecule in cell signaling, and which is considered as a lethal regulator to induce irreversible cellular damage [3].

Anti-oxidative compounds are commonly distributed in our daily diet; these compounds have been extensively studied, aiming to develop health supplements in human [4]. Phenolic compounds are widely distributed in plants, cereals, legumes, nuts, olive oil, vegetables, fruits, traditional Chinese medicine (TCM), tea and red wine [5]. Many phenolic compounds exhibit anti-ROS formation function and possess promising benefits on thrombosis and tumorogenesis [5, 6].

Traditional Chinese medicine (TCM) has been adopted for disease prevention and healing in thousands of years. Herbal decoction, containing different herbs, is one of the effective clinical practices in TCM healing. Decoctions should be prepared by unique methodology according to specific formulation (named as *Fu Fang*) [7]. Based on the syndrome differentiation and the compatible principle of “Master, Minister, Assistant and Servant”, TCM formula should be optimized in order to ease specific syndromes under the guidance of TCM principle [8]. Danggui Buxue Tang (DBT) is a classically herbal decoction utilized for thousands of years, containing Astragali Radix (AR) and Angelica Sinensis Radix (ASR) at the weight ratio 5:1. DBT was recorded in **“*Neiwaishang Bianhuo Lun*”** by “*Li Dongyuan*” in AD1247, in China. Our previous studies demonstrated that there were over fifty components within this herbal decoction [9].

Clinically, DBT was utilized to mitigate cardiovascular illness [10, 11]. Previous study showed that application of DBT in cultured endothelial cells was able to trigger nitric oxide (NO) production [11]. The ingredient responsible for this function was hypothesized to be ferulic acid [12]. Ferulic acid, generated from ASR, is one of the most abundant chemicals within DBT decoction [9, 13]. In line to this notion, ferulic acid showed benefit cardiovascular functions by decreasing the formation of ROS [14, 15]. Thus, ferulic acid could be one key factor to account for anti-oxidative properties of DBT. Here, we would like to address the role of ferulic acid within the herbal mixture by depleting ferulic acid from DBT. This chemically modified DBT decoction was named as DBT_Δfa_. The prevention of ROS formation and the induction of anti-oxidative proteins in cultured H9C2, rat cardiomyoblast cells, were systemically compared between DBT and DBT_Δfa_.

## Materials and Methods

### Plant Materials and Preparation of Herbal Decoctions

Roots of three-year-old *Astragalus memebranaceus* (Fisch.) Bunge var. *mongholicus* (Bunge) Hsiao (AR) from Shanxi Province [16] and two-year-old *Angelica sinensis* (Oliv.) Diel roots (ASR) from Minxian of Gansu Province [17] were harvested in 2013. The authentication of raw materials was identified morphologically by Dr. Tina Dong at The Hong Kong University of Science and Technology (HKUST). The voucher specimens were deposited in the Centre for Chinese Medicine R&D at HKUST. Ferulic acid was purchased from Sigma (St. Louis, MO). Calycosin, formononetin and Z-ligustilide were purchased from TLCM (HKUST, Hong Kong China). The purities of these marker chemicals were higher than 98.0%, which verified by HPLC-DAD. Analytical- and HPLC-grade reagents were from Merck (Darmstadt, Germany).

In order to prepare ferulic acid-depleted DBT (DBT_Δfa_), a Dikma, Diamonsil, C18 column (10.0 mm x 250, 5 μm) was used. The injection volume was 20 μL, and the detection wavelength was set at 254 nm. The mobile phase consisted of acetonitrile (as Solvent A) and 0.01% formic acid (as Solvent B) with a gradient elution programs as follows: 15% to 60% of Solvent A starting from 0 to 70 min. Dividing the ingredients of DBT into two parts: the target compound (ferulic acid) and other compounds (without the target compound). When the elution began, the eluate was collected in one flask. As the ferulic acid peak appeared, the target peak was collected into another flask separately. Once the collection of ferulic acid peak was finished, the residual components were collected in the former flask continuously. Repeating the above procedures, as described above, all the ferulic acid peaks were completely disappeared from the total components peaks. All of the samples generated by chemical-depletion method were lyophilized and re-dissolved in water at 100 mg/mL for biological test and analytic measurement.

### Chemical Fingerprints of DBT

Agilent 1200 series system (Agilent, Waldbronn, Germany), equipped with a degasser, a binary pump, an auto-sampler and a thermo-stated column compartment was used for the analysis. Chromatographic separations were carried out on a Agilent, Eclipse Plus, C18 column (4.6 x 250 mm, 5 μm) with acetonitrile (as solvent A) and 0.01% formic acid (as solvent B) in the mobile phase at a flow rate of 1.0 mL/min at room temperature. The pH of solvent A was 7.0, and the pH of solvent B was 4.7. A liner gradient elution was applied from 15% to 60% of Solvent A starting from 0 to 70 min. Ten μL samples were injected for HPLC analysis and wavelength was set at 254 nm.

### Cell Cultures

H9C2 cell, a cardiomyoblast cell line, was obtained from American Type Culture Collection (ATCC, Manassas, VA). H9C2 cells were cultured in Dulbecco’s modified Eagle’s medium (DMEM), supplemented with 10% fetal bovine serum, 100 units/ml penicillin, and 100 units/ml streptomycin in a humidified CO_2_ (5%) incubator at 37°C [18]. All culture reagents were purchased from Invitrogen Technologies (CarIsbad, CA).

### ROS Formation Assay

The formation of ROS was evaluated using the oxidation-sensitive dye 2’, 7’-dichlorofluorescin diacetate (DCFH-DA) [19]. Cultured cells were pre-treated with tBHQ or herbal extract for 24 hours, then cells were labeled with 10 μM DCFH-DA in HEPES for 1 hour at room temperature. This non-fluorescent dye freely penetrated cells, and then was hydrolyzed to be weak fluorescent, DCFH, which reacted with ROS to form a strong fluorescent signal, DCF. After washing three times with HEPES, the cells were treated with tBHP for 1 hour at room temperature. The amount of intracellular tBHP-induced ROS formation was detected by fluorometric measurement with excitation at 485 nm and emission at 530 nm [19].

### Luciferase Assay

For the transcriptional activation of anti-oxidant response element (ARE), the pARE-Luc (Promega, Fitchburg, WI) DNA construct was employed here. The pGL4.37 (luc2P/ARE/Hygro) vector contains four copies of ARE (5’-TGACnnnGCA-3’) that drives transcription of the luciferase reporter gene *luc2P* (*Photinus pyralis*). The transcriptional factor (Nrf2) binds to ARE for subsequent gene activation. Cultured H9C2 cells were transfected with pARE-Luc by Lipofectamine 3000 (Invitrogen) according to the manufacturer's instructions. H9C2 cells, seeded in 24-well plates, were treated with various concentrations of drugs for 1 day. Then, the medium was aspirated, and the cultures were washed by PBS for twice. The cells were lysed by a buffer containing 0.2% Triton X-100, 1 mM dithiothreitol (DTT) and 100 mM potassium phosphate buffer (pH 7.8) at 4°C. Followed by centrifugation at 13,200 rpm 10 min, the supernatant was collected and used to perform luciferase assay (Tropix Inc., Bedford, MA). The transfection efficiency in H9C2 cells was 40%, as determined by another control plasmid of having a β-galactosidase, under a cytomegalovirus enhancer promoter [19].

### Phosphorylation of Akt

The phosphorylation of Akt (Ser473) was determined by western blot assay. Cultures were serum-starved for 3 hours before the drug or herbal extracts application. After drug treatment, including activators, the cultures were harvested immediately in lysis buffer (125 mM Tris-HCl, 2% SDS, 10% glycerol, 200 mM 2-mercaptoethanol, pH 6.8). Protein concentrations were measured by Bradford’s method (Herculues, CA), and the protein were subjected to SDS-PAGE analysis. After transferring the proteins to membranes, the membranes were incubated with anti-Akt (Upstate, Lake Placid, NY) at 1:5,000 dilution at cold room for overnight. Following incubation in horseradish peroxidase (HRP)-conjugated anti-rabbit secondary antibodies in 1:5,000 dilutions for 3 hours at room temperature, the immune-complexes were visualized by the enhanced chemiluminesence (ECL) method (Amersham Biosciences, Piscataway, NJ). The band intensities in the control and agonist-stimulated samples, run on the same gel and under strictly standardized ECL conditions, were compared on an image analyzer, using in each case a calibration plot constructed from a parallel gel with serial dilutions of one of the samples.

### Protein Expression of NQO1 and GCLM

The protein expressions of NAD(P)H quinone oxidoreductase (NQO1) and glutamate-cysteine ligase catalytic (GCLM) were revealed by western blot. Cultures were seeded onto 6-well plate. After drug treatment for 24 hours, including activators, the cultures were harvested in high salt lysis buffer (1 M NaCl, 10 mM HEPES, pH 7.5, 1 mM EDTA, 0.5% Triton X-100), followed by centrifugation at 16,100 × g for 10 min at 4°C. Samples with equal amount of total protein were added with 2X lysis buffer (0.125 M HCl, pH 6.8, 4% SDS, 20% glycerol, 2% 2-meracptoethanol and 0.02% bromophenol blue) and heated under 95°C, and the protein were subjected to SDS-PAGE analysis. After transferring the proteins to membranes, the membranes were incubated with anti-NQO1 and GCLM antibodies (Upstate, Lake Placid, NY) at 1:5,000 dilution at cold room for overnight. Following incubation in HRP-conjugated anti-rabbit secondary antibodies in 1:5,000 dilutions for 3 hours at room temperature, the immune-complexes were visualized by the ECL method. The band intensities in control and agonist-stimulated samples, run on the same gel and under strictly standardized ECL conditions, were compared on an image analyzer, using in each case a calibration plot constructed from a parallel gel with serial dilutions of one of the samples.

### Statistical Analysis

Principal component analysis (PCA) of the relative peak areas or luciferase reading was performed using SPSS for Windows 16.0 software (SPSS Corporation, Armonk, NY) to evaluate the difference of groups of samples. Data were expressed as the mean ± SEM for *n* = 3 to 5. Statistical tests were performed by one-way ANOVA. In the statistical analyses, differences were classed as significant for values of *p* < 0.05, and highly significant for values of *p* < 0.01 and *p* < 0.001.

## Results

### Chemical Assessments of DBT

**Fig 1:**
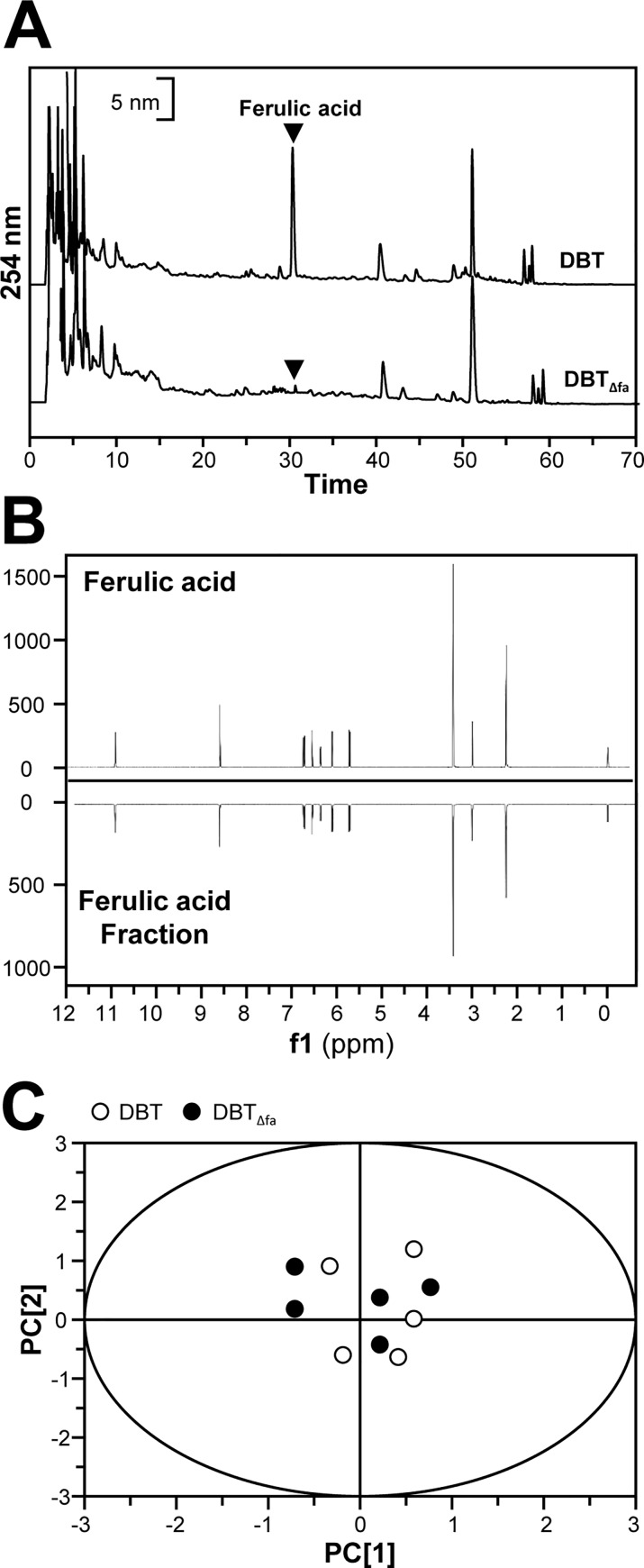
Chemical analysis of DBT and ferulic acid-depleted DBT (DBT_Δfa_). **(A):** Ten μL of 100 mg/mL of parental DBT and DBT_Δfa_ were subjected to HPLC-DAD analysis, and the chemical fingerprints were revealed at the wavelength 254 nm. **(B)**: ^1^H NMR spectra of ferulic acid-isolated fraction, and ferulic acid were revealed. The spectra were recorded in a Varian 600 MHz spectrometer at room temperature. By visual inspection, ferulic acid-isolated fraction showed the similar pattern with ferulic acid. Chemometric analysis by PCA. **(C):** The score plot of peak area of DBT and DBT_Δfa_, determined by HPLC as in (A), was not able to discriminate the difference between authentic DBT and DBT_Δfa_. Representative chromatograms were shown, *n* = 5.

Before performing the biological proprieties of DBT, the first step was to ensure the quality of the decoction. DBT was prepared according to the optimized extraction conditions, as described previously [9, 13, 20]. Four chemicals were selected as markers, including AR-derived calycosin, formononetin, and ASR-derived ferulic acid, Z-ligustilide [7, 16, 18]. **Fig 1A** showed the HPLC fingerprint of DBT at absorbance of 254 nm. A semi-preparative column was employed to create DBT_Δfa_. The fingerprint of DBT_Δfa_ was shown in **Fig 1A**. Ferulic acid peak was depleted from the authentic DBT: this depletion was done by 3 times in order to ensure a complete ferulic acid depletion. After calculating the peak area from HPLC, over 98% of ferulic acid had been removed. The quantitative results were shown in **S1 Table**. Besides, the amount of polysaccharides in DBT was determined by anthrone-sulfuric acid method. DBT contained 13.49% polysaccharides, and DBT_Δfa_ contained 12.98% polysaccharides **(S1 Table).**

The ^1^H NMR metabolic profiling has received attention and served as a quick and generic way for quality control of TCM [21, 22]. Here, we employed ^1^H NMR to reveal the purity of ferulic acid-isolated fraction. From the results, only ferulic acid was identified **(Fig 1B)**. Besides chemical fingerprint, we employed PCA to analyze the difference of DBT and DBT_Δfa._ The main purposes of PCA are to reduce the dimensionality of a dataset. By PCA analysis, we found there were no significant variations of the chemical composition between DBT and DBT_Δfa_ **(Fig 1C)**.

### Oxidant Scavenging Functions of DBT

**Fig 2:**
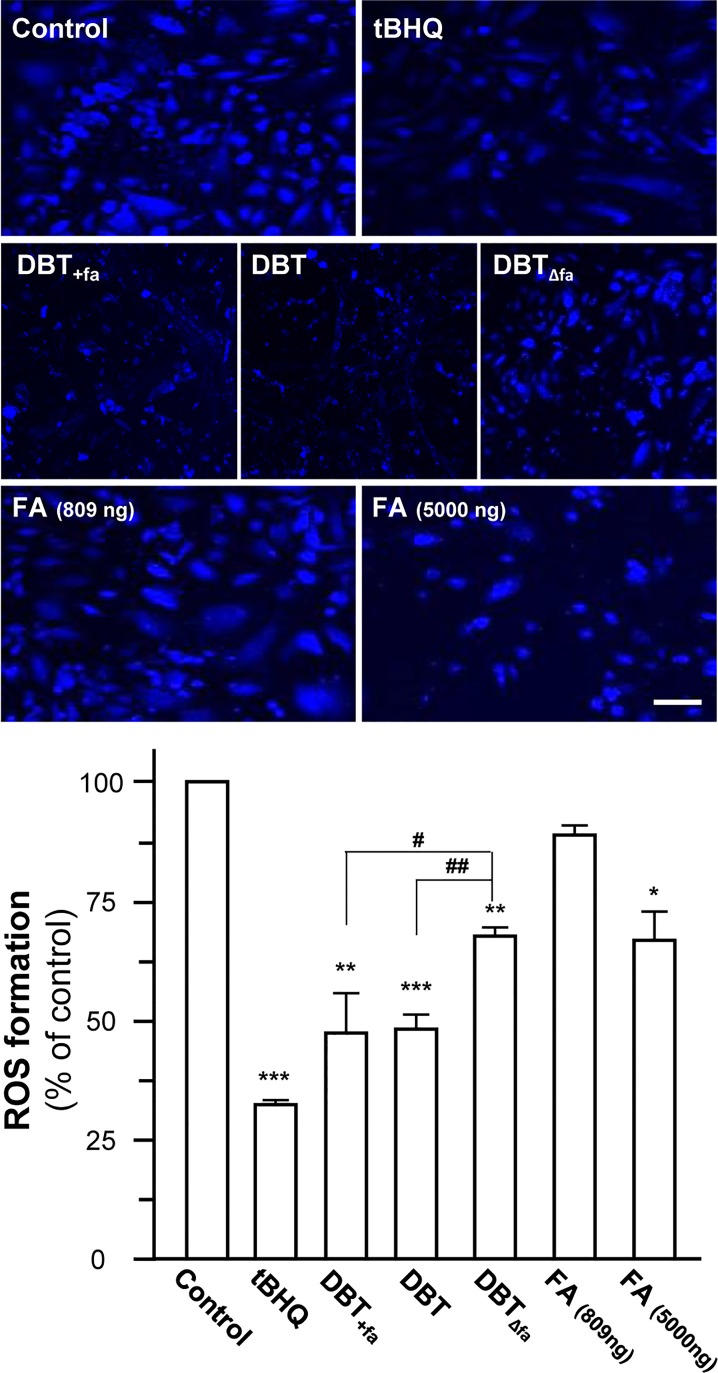
DBT suppresses the tBHP-induced ROS formation. Cultured H9C2 cells were pre-treated with a series of our herbal decoction (1 mg/mL) or ferulic acid (809, 5000 ng/mL) for 24 hours, and then exposed to tBHP (75 μM) for 1 hour. The pre-treatment of tBHQ (5 μM) was used for comparison. The results were in percentage of ROS formation relative to the control (with tBHP alone). Micrographs were taken by a confocal microscope. Bar = 100 μm. Values were expressed as mean ± SEM, where *n* = 3. *** *p* < 0.001, ** *p* < 0.01, * *p* < 0.05, as compared to the control. *# p < 0*.*05*, *## p* < 0.01, as compared to DBT_Δfa_.

The formation of ROS was revealed by laser confocal employing DCF fluorescent dye [19]. The formation of ROS in cultures was in a dose-dependent manner in application of tBHP. tBHQ, a positive control, dramatically decreased the tBHP-induced ROS formation **(Fig 2)**. DBT was able to decrease the generation of tBHP-induced ROS by ~50% **(Fig 2)**. In contrast, the reduction of ROS, induced by DBT_Δfa_, was much lower, as compared with that of the authentic DBT **(Fig 2)**. Ferulic acid by itself at ~800 ng/mL (a concentration being found in 1 mg/mL DBT) did not show effect on tBHP-induced ROS formation, except at a high dose. Similarly, extra ~800 ng ferulic acid in DBT, i.e. DBT_+fa_, did not increase DBT effect in anti-oxidative properties **(Fig 2)**.

**Fig 3:**
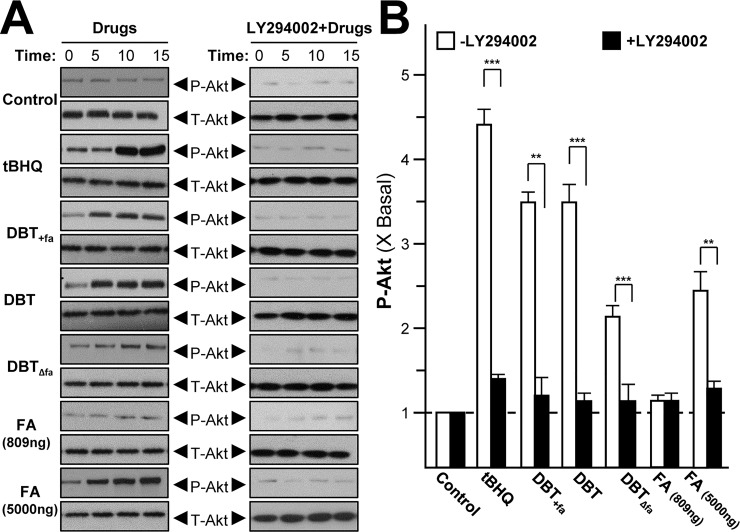
DBT induces the phosphorylation of Akt. Serum free H9C2 cultures were pre-treated with DMEM, or LY294002 at 10 μM, for 3 hours prior to treatment of 1.0 mg/mL of DBT or various concentration of ferulic acid for indicated time. Phosphorylation of Akt (P-Akt) was detected by immunoblot analysis using specific antibodies, total Akt (T-Akt) served as internal control **(A)**. Quantification of P-Akt from the blot at 15 min was calculated by a densitometer **(B)**. Phosphorylation values were expressed as the ratio to the basal reading where the time zero (without treatment) equaled to 1, values were expressed as mean ± SEM, where *n* = 3. *** *p* < 0.001, ** *p* < 0.01 as compared to the control.

The signaling of a serine/threonine-specific protein kinase Akt, also known as protein kinase B (PKB), is reported to play a crucial role in monitoring the survival and apoptosis of cardiomyocytes [23]. Stimulation of Akt has been shown to be vital for cardiovascular protection [13, 24]. Under the stressed condition, the activated form of Akt, i.e. phosphorylated Akt, induces the activated form Nrf2. The active Nrf2 translocates into nucleus and binds to ARE in regulating DNA sequence, and thus which stimulates the expressions of Nrf2-mediated genes, e.g. NQO1 and GCLM. As such, the amount of phospho-Akt is critical to be determined [2]. Ferulic acid-modified DBT was analyzed and compared in cultured H9C2 cells. DBT stimulated the phosphorylation of Akt in a time-dependent manner; the maximal induction was ~3.5 folds at 15 min **(Fig 3A & 3B)**. DBT_Δfa_ showed robust reduction in triggering Akt phosphorylation, at least by 50% reduction. Again, ferulic acid alone showed no effect, except at high concentration. Addition of ferulic acid in DBT, DBT_+fa_, did not increase the phosphorylation. In all cases, the pre-treatment of Akt antagonist, LY294002, dramatically suppressed the activation of Akt **(Fig 3A & 3B)**.

### DBT Induces Anti-Oxidative Enzymes

**Fig 4:**
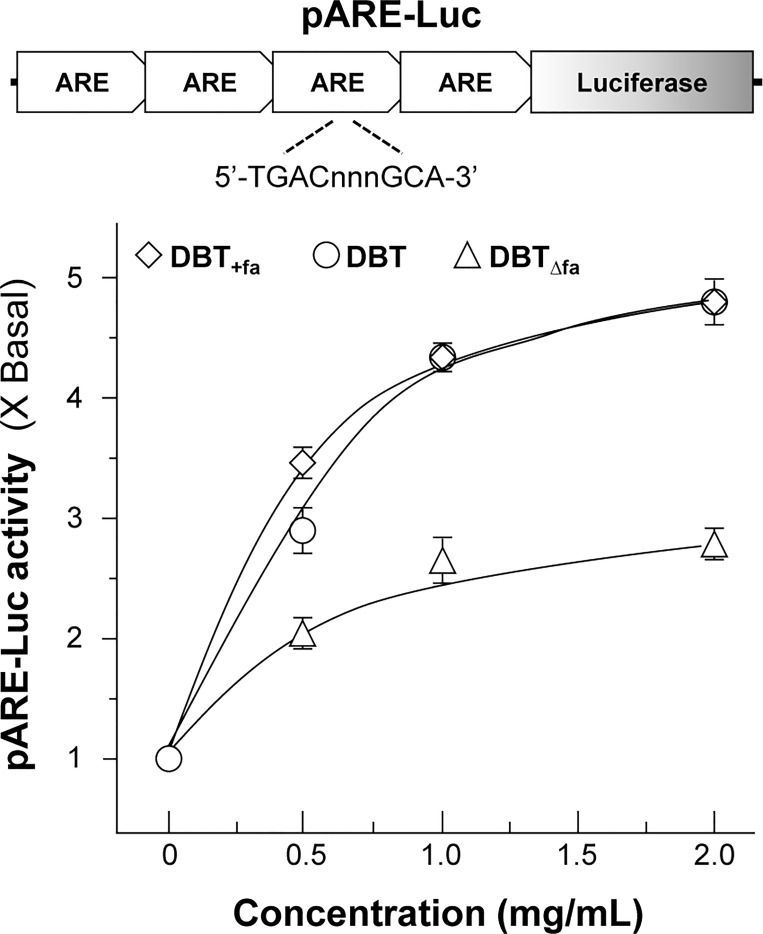
DBT induces the activation of pARE-Luc transfected H9C2 cells. Four repeats of anti-oxidant responsive element (ARE: 5’–TGA Cnn nGC A- 3’) tagged with luciferase reporter vector called pARE-Luc **(upper panel)**. This reporter was stably transfected to H9C2 cells, and which were treated with the herbal decoction for 24 hours **(lower panel)**. tBHQ (5 μM) was used as a positive control of having 5 folds of activation (data not shown). Data were expressed as mean ± SEM, where *n* = 3, each with triplicate samples.

The transcriptional activity of ARE is a crucial regulator against oxidative stress [25]. To explore the function of Akt in ARE transcriptional activation, pARE-Luc construct was transfected into H9C2 cells, and the transcriptional activity of ARE was revealed here. From the results, we found that the stimulation of ARE-driven luciferase activity, induced by the herbal decoctions, showed a dose-dependent manner **(Fig 4)**. The maximal induction of DBT_Δfa_ was ~2.5 folds, which was much weaker than that of authentic DBT or DBT_+fa_ **(Fig 4)**.

**Fig 5:**
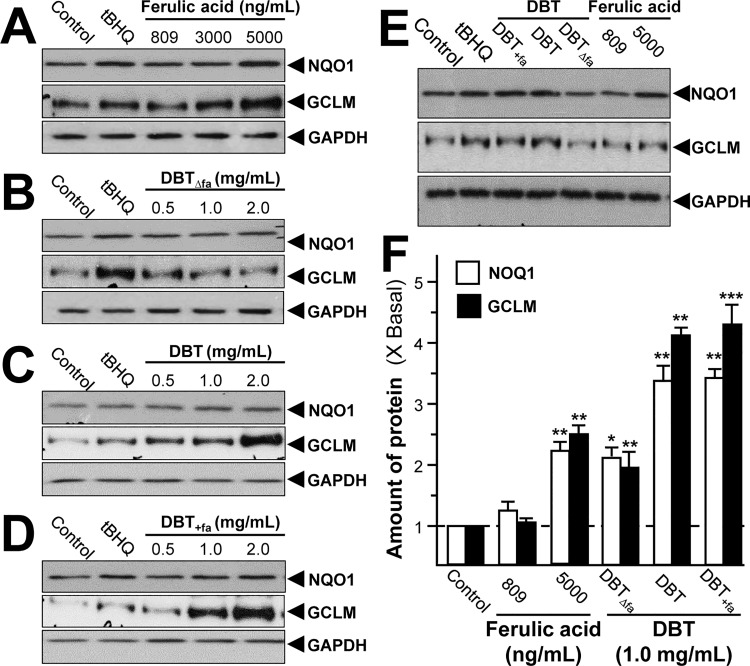
DBT induces the expressions of anti-oxidative enzymes. Cultured H9C2 cells were treated with a series of chemically modified DBT decocton (0.5–2.0 mg/mL) or ferulic acid (809–5000 ng/mL) for 24 hours **(A-E)**. The cell lysates were collected to determine the protein expressions of NQO1 and GCLM using specific antibody. Five μM of tBHQ served as positive control. GAPDH served as loading control. Quantification of protein amount from the blot was calculated by a densitometer (**F**). Values were expressed as the fold of increase to basal reading (untreated culture). Data were expressed as mean ± SEM, where *n* = 3. *** *p* < 0.001, ** *p* < 0.01, * *p* < 0.05 as compared to the control.

Upon activation, ARE stimulates and promotes the transcription of a group of cyto-protective genes [26]. These genes encode enzymes providing anti-oxidants, e.g. NQO1 and GCLM. Here, we revealed the protein levels of NQO1 and GCLM in cultured H9C2 cells after the herbal treatment for 24 hours. Ferulic acid alone did not induce the expressions of NQO1 and GCLM, except at high concentration **(Fig 5)**. DBT, as well as DBT_+fa_, induced the protein expressions about 3 to 4 folds. Again, DBT_Δfa_ reduced the potency of DBT in inducing the protein expressions **(Fig 5)**.

**Fig 6:**
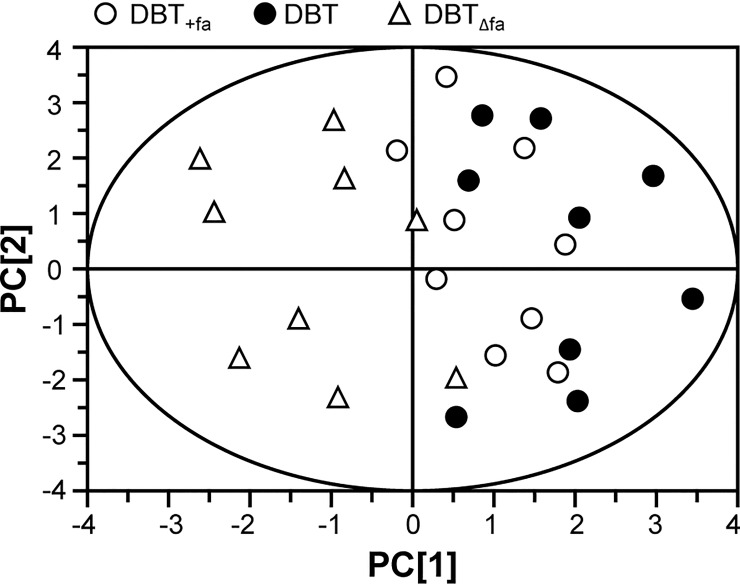
PCA analysis of DBT, DBT_Δfa_ and DBT_+fa_. The score plot of biological properties from various ferulic acid-modified DBT. The input materials were the luciferase data at 1 mg/mL of DBT, the phosphorylation values at 15 min, and the protein levels of NQO1 and GCLM. The depletion of ferulic acid in DBT, i.e. DBT_Δfa_ showed a significant effect on the bioactivities. Addition of ferulic acid in DBT, DBT_+fa_, did not affect the properties.

PCA provides a roadmap to show how a complex data set can be transformed to a lower dimension in order to reveal the differences of various samples [21, 22]. The loading plots of PCA reveal whether the biological data contribute significantly to the intergroup differences in which they are farthest from the main cluster of analyzed materials. The input materials were the luciferase data at 1 mg/mL, the phosphorylation values at 15 min and the protein levels of NQO1 and GCLM, as induced by our herbal decoctions. As a result, these biological properties could be significant for determining DBT or DBT_+fa_ from DBT_Δfa_ **(Fig 6)**.

## Discussion

ROS is being produced in responding to many hormones, e.g. auxin, abscisic acid and salicylic acid. Thus, ROS is a key factor in sensing and responding to environmental changes, as well as in orchestrating human body responses and development [27]. The harmful functions of ROS come in a form of DNA damage, lipid preoccupation, protein amino acid oxidation, and in activation of specific enzymes by oxidation of their cofactors. These damages lead to many specific or general illnesses and problems, such as Keshan disease, stroke, neurotoxins, cancer, and renal graft glomerulonephritis [28]. Besides, ROS is accumulated with age [29], which is proposed to play roles in age-related illnesses, i.e. cancer and cardiovascular illness [30]. Considerable data showed that ROS was a critical regulator inducing cardiovascular diseases [31]. However, numerous approaches of western medicine for mitigating cardiovascular disease have not received favorable results [32].

Herbal decoction has numerous ingredients, and indeed the role of individual chemical is hard to identify. Chemical knock-out method is one of the approaches being used to reveal the action mechanism of DBT. Previously, we have generated calycosin-depleted DBT, i.e. DBT_Δcal_, and this chemically modified herbal decoction lost its functions in regulating estrogenic erythropoietic, osteogenic properties in different cells [13]. Similarly, calycosin alone did not show any functions of that of DBT. Here, we demonstrated the anti-oxidative properties of DBT could be mainly mediated by ferulic acid. However, ferulic acid alone was not able to trigger the DBT activities, at least in a dose that was corresponding to DBT at 1 mg/mL, which suggested that ferulic acid played an important role in orchestrating anti-oxidative functions of DBT. We hypothesized that those key chemicals, e.g. calycosin and ferulic acid, act as organizers in triggering various functions of a herbal mixture. However, DBT_Δfa_ did not affect estrogenic, osteogenic and erythropoietic properties **(S1 Fig)**, while DBT_Δcal_ did not affect anti-oxidative properties in cultured H9C2 cells **(S2 Fig)**. Thus, this chemical knock-out methodology could be used for other herbal mixture, as to reveal its action mechanism.

ASR has been utilized for thousands of years, and this is widely utilized for treating various illness [33]. Previous papers showed that ASR possessed anti-oxidative effects, promoted angiogenesis, prevented chronic cardiotoxicity and reduced myocardial injury *in vivo* [34, 35]. Besides the role of ASR in DBT, ASR plays key functional roles in various herbal mixtures. For examples, Buzhongyiqi decoction, a popular herbal formula, contains 9 different herbs including ASR, which has been utilized for easing oxidative stress induced diseases [36]. The *in vivo* study of Buzhongyiqi decoction presented that this herbal decoction reduced myocardial lesion, improved heart antioxidant capacity and suppressed the cardiomyocyte apoptosis [37]. Huang et al., (2014) demonstrated that ASR attenuated angiotensin II-induced cell death in H9C2 cells through JNK and PI3K inhibitions [33]. The major ingredients of ASR were reported to be Z-ligustilide and ferulic acid [11, 18, 37]. Ferulic acid was shown to increase blood fluidity, to inhibit platelet aggregation *in vivo*, to decrease serum lipids, to prevent thrombus formation, to protect neurons, to exhibit anti-cancer and anti-oxidative functions [38]. Moreover, ferulic acid had anti-inflammatory functions, prevented liver injury, restricted viral infections, inhibited production of interleukin-8, and harmonized various inflammatory reactions in human body [38, 39]. After oral administration of DBT in rat, the ferulic acid was found in the plasma, which suggested the bioavailability of ferulic acid was shown to be ~10% [40]. Keap1-Nrf2-ARE is an important signaling pathway to modulate the anti-oxidative functions [25]. Under unstressed conditions, Nrf2 acts as an inactive complex with its repressor Keap1, which negatively modulates Nrf2 by ubiquitination and proteasomal degradation [25]. Our data suggested that DBT modulated the anti-oxidative effects through the Keap1-Nrf2-ARE mechanism. Furthermore, DBT_Δfa_ exhibited much lower activation inducing the protein expressions of NQO1 and GCLM, transcriptional activity of ARE, and phosphorylation of Akt. All of the results suggested that ferulic acid played an indispensable role in DBT decoction in terms of protecting injury of cardiomyocyte.

## Supporting Information

S1 Fig Reduced amount of ferulic acid does not alter estrogenic, osteogenic and erythropoietic functions on different cell models. **(A):** In pERE-Luc stably transfected MCF-7 cultures, DBT and DBT_Δfa_ were applied for 48 hours at different concentrations (0.125–2.0 mg/mL). Cell lysate were subjected to the luciferase assay. 17-Estrodiol (E_2_; 100 nM) served as a positive control, which caused ~1.4-fold increase of the luciferase **(B):** Water extracts of DBT or DBT_Δfa_ at different concentrations (0.125–2.0 mg/mL) were applied onto cultured MG-63 cells for 48 hours before the analysis on the enzymatic activity of ALP. Dexamethasone (50 nM) together with vitamin C (250 μM) was used as positive control in MG-63 cell, which activated the ALP activity by ~1.8-fold. **(C):** Cultured Hep3B cells were transfected with pHRE-Luc construct. The pHRE-Luc expressed cells were treated with a series concentrations of DBT decoction (0.5–2.0 mg/mL) for 48 hours. The cell lysates were subjected to luciferase assay.Values were shown in fold of changes as compared to control. Values were in mean ± SEM, *n* = 3, each with triplicate.
(TIF)

S2 Fig DBT_Δcal_ and authentic DBT have similar roles in triggering anti-oxidative functions. Cultured H9C2 cells were treated with herbal extracts (1.0 mg/mL) for 24 hours. The cell lysates were collected to determine the protein expressions of NQO1 and GCLM by specific antibody. Five μM of tBHQ served as positive control. GAPDH served as loading control. Quantification of protein from the blot was calculated by a densitometer. Values were expressed as the fold of increase to basal reading (untreated culture). Data were expressed as mean ± SEM, where *n* = 3. *** *p* < 0.001, ** *p* < 0.01 as compared to the control.
(TIF)

S1 Table Quantitative assessment of marker chemicals in DBT, DBT_Δfa_ and the collected ferulic acid fraction. ^a^ Four markers were selected as marker chemicals, and which were determined by HPLC method. These chemicals set parameters for minimal requirement for the quality control. ^b^ DBT_Δfa_ generated as stated in the method. ^c^ The ferulic acid fraction was collected from the preparative HPLC of DBT, as stated in method. ^d^ Values were expressed in μg/g dried extract of DBT, in Mean ± SEM, where *n* = 3. ^e^ Not detected. ^f^ The content of polysaccharides was determined by anthrone-sulfuric acid method. Values were expressed in mean ± SEM, where *n* = 3. *** *p* < 0.001 as compared with authentic DBT.
(DOCX)

## Abbreviations

DBT: Danggui Buxue Tang
AR: Astragali Radix
ARE: Anti-oxidant response element
ROS: Reactive oxygen species
TCM: Traditional Chinese medicine
ASR: Angelica Sinensis Radix
NO: Nitric oxide
ECL: Chemiluminesence

## References

[1] Cutler D, Deaton A, Lleras-Muney A. The determinants of mortality. J Econ Perspect 2006; 20: 97–102.

[2] Giugliano D. Dietary antioxidants for cardiovascular prevention. Nutr Metab Cardiovasc Dis 2000; 10: 38–44. 10812586

[3] Fang B, Xiao HJ. Rapamycin alleviates cisplatin-induced ototoxicity *in vivo*. Biochem Biophys Res Commun 2014; 448:443–447. 10.1016/j.bbrc.2014.04.123 24796670

[4] Carlsen MH, Halvorsen BL, Holte K, Bøhn SK, Dragland S, Sampson L, et al The total antioxidant content of more than 3100 foods, beverages, spices, herbs and supplements used worldwide. Nutr J 2010; 9: 3 10.1186/1475-2891-9-3 20096093 PMC2841576

[5] Kris-Etherton PM, Hecker KD, Bonanome A, Coval SM, Binkoski AE, Hilpert KF, et al Bioactive compounds in foods: their role in the prevention of cardiovascular disease and cancer. Am J Med 2002; 113: 71S–88S. 12566142 10.1016/s0002-9343(01)00995-0

[6] Yu CL, Zhao XM, Niu YC. Ferulic acid protects against lead acetate-induced inhibition of neurite outgrowth by upregulating HO-1 in PC12 cells: involvement of ERK1/2-Nrf2 pathway. Mol Neurobiol 2015; 10.1007/s12035-015-9555-x 26611834

[7] Tan Y, Li J, Lu C, He XJ, Jiang M, Lu AP. Modern elucidative strategies for scientific connotation of controlling toxic reactions while toxic herbs are used to the indication syndrome. Zhongguo Zhong Xi Yi Jie He Za Zhi 2013; 33: 1412–1415. 24432691

[8] Chen K, Yu B. Certain progress of clinical research on Chinese integrative medicine. Chin Med 1999; 112: 934–937. 11717980

[9] Dong TTX, Zhao KJ, Gao QT, Ji ZN, Zhu TT, Li J, et al Chemical and biological assessment of a Chinese herbal decoction containing Radix Astragali and Radix Angelicae Sinensis: determination of drug ratio in having optimized properties. J Agric Food Chem 2006; 54, 2767–2774. 10.1021/jf053163l 16569074

[10] Mak DH, Chiu PY, Dong TT, Tsim KW, Ko KM. Dang-Gui Buxue Tang produces a more potent cardioprotective effect than its component herb extracts and enhances glutathione status in rat heart mitochondria and erythrocytes. Phytother Res 2006; 20: 561–567. 10.1002/ptr.1904 16619337

[11] Gong AG, Lau KM, Zhang LM, Lin HQ, Dong TT, Tsim KW. Danggui Buxue Tang, Chinese herbal decoction containing Astragali Radix and Angelicae Sinensis Radix, induces production of nitric oxide in endothelial cells: signaling mediated by phosphorylation of endothelial nitric oxide synthase. Planta Med 2016; 82: 418–423. 10.1055/s-0035-1558332 26824621

[12] Li X, Wu X, Huang L. Correlation between antioxidant activities and phenolic contents of Radix Angelicae Sinensis (Danggui). Molecules 2009; 14: 5349–5361. 10.3390/molecules14125349 20032898 PMC6255375

[13] Gong AGW, Li N, Lau KM, Lee PSC, Yan L, Xu ML, et al Calycosin orchestrates the functions of Danggui Buxue Tang, a Chinese herbal decoction composing of Astragali Radix and Angelica Sinensis Radix: an evaluation by using calycosin-knock out herbal extract. J Ethnopharmacol 2015; 168, 150–157. 10.1016/j.jep.2015.03.033 25796405

[14] Alam B, Akter F, Parvin N, Sharmin PR, Akter S, Chowdhury J. Antioxidant, analgesic and anti-inflammatory activities of the methanolic extract of Piper Betle leaves. Avicenna J Phytomed 2013; 3: 112–125. 25050265 PMC4075698

[15] Kumar SV, Anders HJ. Glomerular disease: limiting autoimmune tissue injury: ROS and the inflammasome. Nat Rev Nephrol 2014; 10: 545–546. 10.1038/nrneph.2014.156 25157839

[16] Ma XQ, Shi Q, Duan JA, Dong TTX, Tsim KWK. Chemical analysis of Radix Astragali (Huangqi) in China: a comparison with its adulterants and seasonal variations. J Agric Food Chem 2002; 50: 4861–4866. 12166972 10.1021/jf0202279

[17] Zhao KJ, Dong TTX, Tu PF, Song ZH, Lo CK, Tsim KWK. Molecular genetics and chemical assessment of Radix Angelica (Danggui) in China. J Agric Food Chem 2003; 51, 2576–2583. 10.1021/jf026178h 12696940

[18] Zhang WL, Choi RC, Zhan JY, Chen JP, Luk WK, Yao P, et al Can Hedysari Radix replace Astragali Radix in Danggui Buxue Tang, a Chinese herbal decoction for woman aliment? Phytomedicine 2013; 20, 1076–1081. 10.1016/j.phymed.2013.04.016 23746954

[19] Maiwulanjiang M, Chen J, Xin G, Gong AG, Miernisha A, Du CY, et al The volatile oil of Nardostachyos Radix et Rhizoma inhibits the oxidative stress-induced cell injury via reactive oxygen species scavenging and Akt activation in H9C2 cardiomyocyte. J Ethnopharmacol 2014; 153: 491–498. 10.1016/j.jep.2014.03.010 24632018

[20] Song ZH, Ji ZN, Lo CK, Dong TT, Zhao KJ, Li OT, et al Chemical and biological assessment of a traditional Chinese herbal decoction prepared from Radix Astragali and Radix Angelicae Sinensis: orthogonal array design to optimize the extraction of chemical constituents. Planta Med 2014; 70: 1222–1227. 10.1055/s-2004-835855 15643561

[21] Zhan JY, Zheng KY, Zhu KY, Zhang WL, Bi CW, Chen JP, et al Importance of wine-treated Angelica Sinensis Radix in Si Wu Tang, a traditional herbal formula for treating women's ailments. Planta Med 2013; 79: 533–537. 10.1055/s-0032-1328261 23457023

[22] Chan PH, Zhang WL, Cheung CY, Tsim KW, Lam H. Quality control of Danggui Buxue Tang, a traditional Chinese medicine decoction, by (1)H-NMR metabolic profiling. Evid Based Complement Alternat Med 2014; 567893 10.1155/2014/567893 24826194 PMC3980871

[23] Jun HO, Kim DH, Lee SW, Lee HS, Seo JH, Kim JH, et al Clusterin protects H9C2 cardiomyocytes from oxidative stress-induced apoptosis via Akt/GSK-3β signaling pathway. Exp Mol Med 2011; 43: 53–61. 10.3858/emm.2011.43.1.006 21270507 PMC3041938

[24] Pachori AS, Smith A, McDonald P, Zhang L, Dzau VJ, Melo LG. Heme- oxygenase-1-induced protection against hypoxia/reoxygenation is dependent on biliverdin reductase and its interaction with PI3K/Akt pathway. J Mol Cell Cardiol 2007; 43: 580–592. 10.1016/j.yjmcc.2007.08.003 17920074 PMC2699998

[25] Nguyen T, Nioi P, Pickett CB. The Nrf2-antioxidant response element signaling pathway and its activation by oxidative stress. J Biol Chem 2009; 284: 13291–13295. 10.1074/jbc.R900010200 19182219 PMC2679427

[26] Joung EJ, Li MH, Lee HG, Somparn N, Jung YS, Na HK, et al Capsaicin induces heme oxygenase-1 expression in HepG2 cells via activation of PI3K-Nrf2 signaling: NAD(P)H: quinone oxidoreductase as a potential target. Antioxid Redox Signal 2007; 9: 2087–2098. 10.1089/ars.2007.1827 17979524

[27] Filomeni G, Zio DD, Cecconi F. Oxidative stress and autophagy: the clash between damage and metabolic needs. Cell Death Differ 2015; 22: 377–288. 10.1038/cdd.2014.150 25257172 PMC4326572

[28] Cai ZY, Yan LJ. Protein oxidative modifications: beneficial roles in disease and health. J Biochem Pharmacol Res 2013; 1: 15–26. 23662248 PMC3646577

[29] Gan XT, Hunter JC, Huang C, Xue J, Rajapurohitam V, Javadov S, et al 2012. Ouabain increases iNOS-dependent nitric oxide generation which contributes to the hypertrophic effect of the glycoside: possible role of peroxynitrite formation. Mol Cell Biochem 2012; 363: 323–333. 10.1007/s11010-011-1185-7 22160804

[30] Uttara B, Singh AV, Zamboni P, Mahajan RT. Oxidative stress and neurodegenerative diseases: a review of upstream and downstream antioxidant therapeutic options. Curr Neuropharmacol 2009; 7: 65–74. 10.2174/157015909787602823 19721819 PMC2724665

[31] Sugamura K, Keaney JF. Reactive oxygen species in cardiovascular disease. Free Radic Biol Med 2011; 51: 978–992. 10.1016/j.freeradbiomed.2011.05.004 21627987 PMC3156326

[32] Li SM, Xu H. Integrative western and Chinese medicine on coronary heart disease: where is the orientation? Evid Based Complement Alternat Med 2013: 459264 10.1155/2013/459264 24023575 PMC3760124

[33] Huang CY, Kuo WW, Kuo CH, Tsai FJ, Liu PY, Hsieh DJ. Protective effect of Danggui (Radix Angelicae Sinensis) on angiotensin II-induced apoptosis in H9C2 cardiomyoblast cells. BMC Complement Altern Med 2014; 14: 358 10.1186/1472-6882-14-358 25256260 PMC4182826

[34] Xin YF, Zhou GL, Shen M, Chen YX, Liu SP, Chen GC, et al *Angelica Sinensis*: a novel adjunct to prevent doxorubicin-induced chronic cardiotoxicity. Basic Clin Pharmacol Toxicol 2007; 101: 421–426. 10.1111/j.1742-7843.2007.00144.x 17971065

[35] Zhang A, Sun H, Wang Z, Sun W, Wang P, Wang X. Metabolomics: towards understanding traditional Chinese medicine. Planta Med 2010; 76: 2026–2035. 10.1055/s-0030-1250542 21058239

[36] Cui L, Wang Y, Liu Z, Chen H, Wang H, Zhou X, et al Discovering New Acetylcholinesterase inhibitors by mining the Buzhongyiqi decoction recipe data. J Chem Inf Model 2015; 55: 2455–2463. 10.1021/acs.jcim.5b00449 26509353

[37] Wang XL, Wang T, Wang YN. Effect of Danggui Buxue decoction on the hemopoiesis reconstruction of mouse transplanted by the muscle satellite cell receptor. Zhongguo Zhong Xi Yi Jie He Za Zhi 2011; 31: 1093–1096. 21910342

[38] Hou YZ, Yang J, Zhao GR, Yuan YJ. Ferulic acid inhibits vascular smooth muscle cell proliferation induced by angiotensin II. Eur J Pharmacol 2004; 499: 85–90. 10.1016/j.ejphar.2004.07.107 15363954

[39] Mathew S, Abraham TE. Ferulic acid: an antioxidant found naturally in plant cell walls and feruloyl esterases involved in its release and their applications. Crit Rev Biotechnol 2004; 24: 59–83. 10.1080/07388550490491467 15493526

[40] Wen XD, Qi LW, Li P, Bao KD, Yan XW, Yi L, Li CY. Simultaneous determination of calycosin-7-O-β-d-glucoside, ononin, astragaloside IV, astragaloside I and ferulic acid in rat plasma after oral administration of Danggui Buxue Tang extract for their pharmacokinetic studies by liquid chromatography-mass spectrometry J Chromatogr B 2008; 865: 99–105. 10.1016/j.jchromb.2008.02.024 18346946

