# Supplementary figures and images for: Ferulic Acid Orchestrates Anti-Oxidative Properties of Danggui Buxue Tang, an Ancient Herbal Decoction: Elucidation by Chemical Knock-Out Approach
Source: PLoS One. 2016 Nov 8;11(11):e0165486. doi: 10.1371/journal.pone.0165486 (PMC5100993; doi:10.1371/journal.pone.0165486)

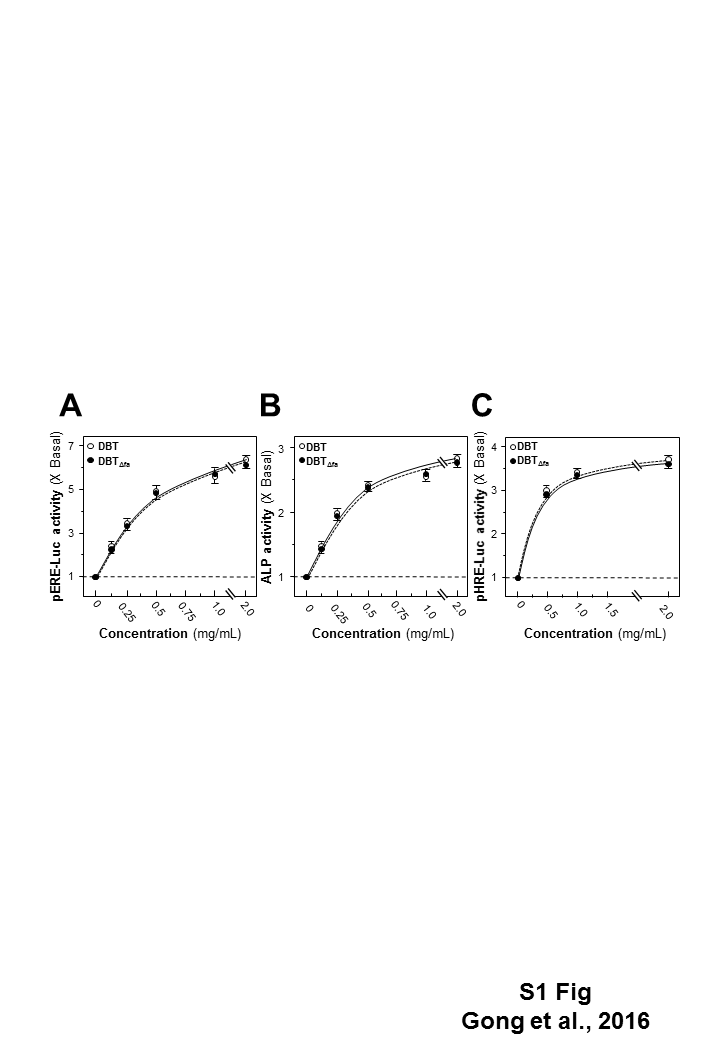

Supplement: S1 Fig — (A): In pERE-Luc stably transfected MCF-7 cultures, DBT and DBTΔfa were applied for 48 hours at different concentrations (0.125–2.0 mg/mL). Cell lysate were subjected to the luciferase assay. 17-Estrodiol (E2; 100 nM) served as a positive control, which caused ~1.4-fold increase of the luciferase (B): Water extracts of DBT or DBTΔfa at different concentrations (0.125–2.0 mg/mL) were applied onto cultured MG-63 cells for 48 hours before the analysis on the enzymatic activity of ALP. Dexamethasone (50 nM) together with vitamin C (250 μM) was used as positive control in MG-63 cell, which activated the ALP activity by ~1.8-fold. (C): Cultured Hep3B cells were transfected with pHRE-Luc construct. The pHRE-Luc expressed cells were treated with a series concentrations of DBT decoction (0.5–2.0 mg/mL) for 48 hours. The cell lysates were subjected to luciferase assay.Values were shown in fold of changes as compared to control. Values were in mean ± SEM, n = 3, each with triplicate. (TIF) [file pone.0165486.s001.TIF]

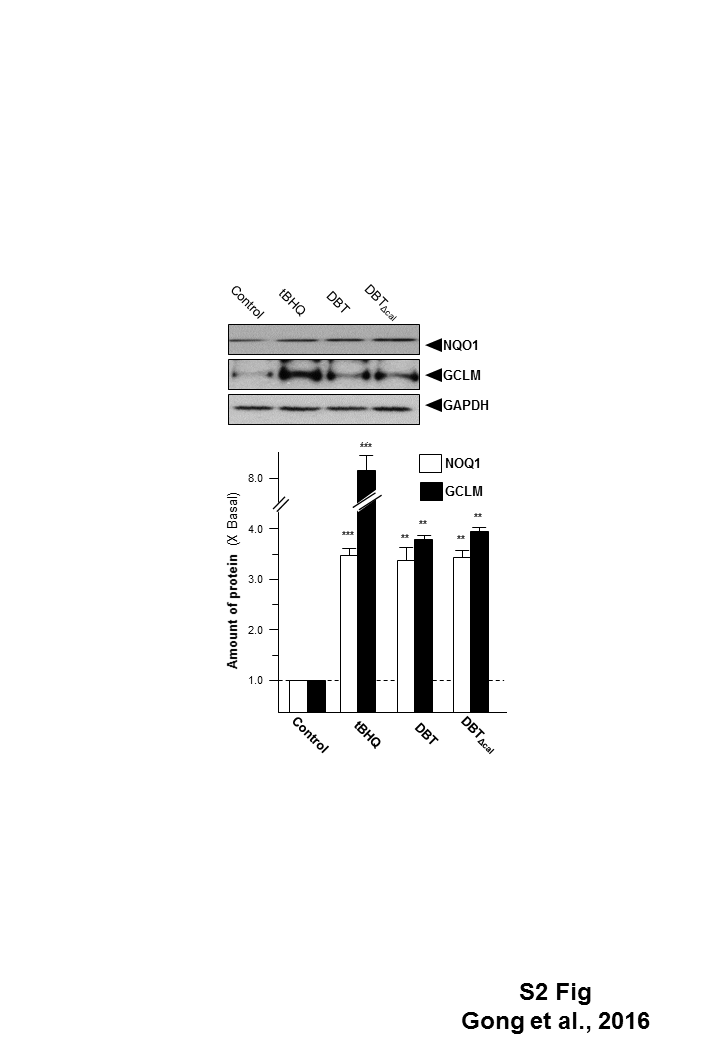

Supplement: S2 Fig — Cultured H9C2 cells were treated with herbal extracts (1.0 mg/mL) for 24 hours. The cell lysates were collected to determine the protein expressions of NQO1 and GCLM by specific antibody. Five μM of tBHQ served as positive control. GAPDH served as loading control. Quantification of protein from the blot was calculated by a densitometer. Values were expressed as the fold of increase to basal reading (untreated culture). Data were expressed as mean ± SEM, where n = 3. *** p < 0.001, ** p < 0.01 as compared to the control. (TIF) [file pone.0165486.s002.TIF]
