# Supplementary material for: Ferulic Acid Orchestrates Anti-Oxidative Properties of Danggui Buxue Tang, an Ancient Herbal Decoction: Elucidation by Chemical Knock-Out Approach
Source: PLoS One. 2016 Nov 8;11(11):e0165486. doi: 10.1371/journal.pone.0165486 (PMC5100993; doi:10.1371/journal.pone.0165486)
Supplement: S1 Table — a Four markers were selected as marker chemicals, and which were determined by HPLC method. These chemicals set parameters for minimal requirement for the quality control. b DBTΔfa generated as stated in the method. c The ferulic acid fraction was collected from the preparative HPLC of DBT, as stated in method. d Values were expressed in μg/g dried extract of DBT, in Mean ± SEM, where n = 3. e Not detected. f The content of polysaccharides was determined by anthrone-sulfuric acid method. Values were expressed in mean ± SEM, where n = 3. *** p < 0.001 as compared with authentic DBT. (DOCX) [file pone.0165486.s003.docx]

**S1 Table.** Quantitative assessment of marker chemicals in DBT, DBT_∆fa_ and the collected ferulic acid fraction

| Chemical^a^ | Sample | | |  |
| --- | --- | --- | --- | --- |
|  | DBT | DBT_∆fa_^b^ | Ferulic acid fraction^c^ |  |
| **Ferulic acid** | 809.56 ± 2.00^d^ | 12.14 ± 1.48**^***^** | 798.33 ± 2.65 |  |
| **Calycosin** | 693.22 ± 1.79 | 692.05 ± 1.33 | -^e^ |  |
| **Formononetin** | 163.90 ± 4.25 | 164.12 ± 3.11 | - |  |
| **Z-ligustilide** | 212.35 ± 4.62 | 212.73 ± 3.98 | - |  |
| **Polysaccharides** | 13.49 ± 0.53^f^ | 12.98 ± 2.01 | - |  |

^a^ Four markers were selected as marker chemicals, and which were determined by HPLC method. These chemicals set parameters for minimal requirement for the quality control.

^b^ DBT_∆fa_ generated as stated in the method.

^c^ The ferulic acid fraction was collected from the preparative HPLC of DBT, as stated in method.

^d^ Values were expressed in µg/g dried extract of DBT, in Mean ± SEM, where *n* = 3.

*** *p* < 0.001 as compared with authentic DBT.

^e^ Not detected.

^f^ The content of polysaccharides was determined by anthrone-sulfuric acid method. Values were expressed in mean ± SEM, where *n* = 3.
